# Health and Social Problems of the Elderly: A Cross-Sectional Study in Udupi Taluk, Karnataka

**DOI:** 10.4103/0970-0218.51236

**Published:** 2009-04

**Authors:** A Lena, K Ashok, M Padma, V Kamath, A Kamath

**Affiliations:** Department of Community Medicine, Kasturba Medical College, Manipal, India; 1 Department of Community Medicine, Kasturba Medical College, Mangalore, India

**Keywords:** Attitude, elderly, morbidity, social and health problems

## Abstract

**Background:**

Change in socio-economic status and various health problems adversely affect an individual's way of life during old age.

**Objectives:**

To study the health and social problems of the elderly and their attitude towards life.

**Materials and Methods:**

Descriptive study carried out in the Field practice area of the Department of Community Medicine in South India. A total of 213 elderly patients (60 years old and above) who attended the outreach clinics were interviewed using a pre-tested schedule. Findings were described in terms of proportions and percentages to study the socio-economic status of the samples and its correlation to social problems.

**Results:**

Around 73% of the patients belonged to the age group of 60-69 years old. Nearly half of the respondents were illiterate. Around 48% felt they were not happy in life. A majority of them had health problems such as hypertension followed by arthritis, diabetes, asthma, cataract, and anemia. About 68% of the patients said that the attitude of people towards the elderly was that of neglect.

**Conclusions:**

The results of the study showed that there is a need for geriatric counseling centers that can take care of their physical and psychological needs. The stringent rules for eligibility to social security schemes should be made more flexible to cover a larger population.

## Introduction

There is no United Nations standard numerical criterion, but the UN agreed cutoff is 60+ years when referring to the elderly population.(1) In India, the elderly account for 7% of the total population, of which two-thirds live in villages and nearly half of them in poor conditions.(2) Urbanisation, nuclearisation of family, migration, and dual career(3) families are making care of the elderly more and more of a personal and social problem in India.

With the decline in fertility and mortality rates accompanied by an improvement in child survival and increased life expectancy, a significant feature of demographic change is the progressive increase in the number of elderly persons. Increasing life span and poor health care add to the degree of disability among the elderly and compound the problems of care giving. In India, the life expectancy has steadily gone up from 32 years at the time of independence to over 63 in 2001. The elderly experience changes in different aspects of their lives.

The physiological decline in ageing refers to the physical changes an individual experiences because of the decline in the normal functioning of the body resulting in poor mobility, vision, hearing, inability to eat and digest food properly, a decline in memory, the inability to control certain physiological functions, and various chronic conditions. Change in socio-economic status adversely affects the individual's way of life after retirement. The economic loss is due to a change from salary to pension or unemployment leading to economic dependency on children or relatives. A feeling of low self-worth may be felt due to the loss of earning power and social recognition. This state of mind is harmful. With the prospect of this situation worsening in the coming decades, ways and means of managing the stress effectively needs to be examined.(4)

This study was thus conducted with the following objectives:

1. To study the background and socio-economic status of the elderly
2. To study the social and health problems faced by the elderly and their attitude towards life

## Materials and Methods

This study was carried out over a period of 1 year from January to December 2003. The study subjects included elderly men and women aged 60 years and above(1,5,6) who belonged to the rural field practice area of the Department of Community Medicine, a Medical College located in South India.

The field practice area has six rural maternity and child welfare centers covering 50,000 people. Clinics for the general population and women and children are regularly held in these centers by the department.

The subjects for this study were the elderly patients attending these clinics regularly for various health problems. The questionnaire was developed by reviewing related Indian studies. This questionnaire was then pilot tested on ten elderly individuals and the necessary changes were made.

A total of 213 subjects were interviewed using this pre-tested questionnaire by a trained health educator who was one of the investigators in this study. The interview was carried out in the local language. The purpose of the study was explained to them and oral informed consent was obtained. Care was also taken to ensure privacy and confidentiality of the interview as part of the study. In order to avoid the interference and influence of other family members and neighbors, each respondent was called and interviewed privately where he/she could feel comfortable. The data collected was tabulated and analysed using the statistical package SPSS, Version 11.5 for Windows™. Findings were described using proportions and percentages.

## Results

### Socio-demographic characteristics

Table 1 shows that a major fraction of the population was in the age group of 60-69 years old, while a small fraction (2.8%) were 80 years old or older. Males and females formed an almost equal proportion of the study sample. A majority (89%) of the respondents were Hindus. This reflects the true picture of the population based on religion at the local and national level. A joint family system was seen to be the most common (56.8%) among the population interviewed followed by the nuclear family. Only 12.1% of the elderly men were widowed while 67.7% of the women were widows. The unmarried group of 2.3% was comprised of only men. Literacy was found to be low in the study population.

**Table 1:** Demographic distribution of the respondents

	Males	Females	Total (n=213)
Age (years)			
60 - 69	57 (61.9)	97 (80.1)	154 (72.3)
70 - 79	31 (33.7)	22 (18.2)	53 (24.8)
>80	4 (4.3)	2 (1.7)	6 (2.8)
Marital status			
Married	70 (76.1)	31 (25.6)	101 (47.4)
Single	5 (5.4)	0 (0)	5 (2.3)
Separated	6 (6.5)	8 (6.6)	14 (6.6)
Widow/Widower	11 (12.1)	82 (67.7)	93 (79.8)
Education			
Illiterate	21 (22.8)	75 (62)	96 (45.1)
Just literate	1 (1.1)	1 (0.8)	2 (0.9)
Primary	40 (43.5)	38 (31.4)	78 (36.6)
Secondary	14 (15.2)	5 (4.1)	19 (8.9)
High school	13 (14.1)	2 (1.7)	15 (7.0)
Intermediate	2 (2.2)	0 (0)	2 (0.9)
Graduate	1 (1.1)	0 (0)	1 (0.5)

Figures in parentheses are in percentages

### Health problems of the elderly

Table 2 shows that all the respondents had health problems, the most common being hypertension, osteoarthritis, diabetes, or bronchial asthma. Others included cataract, anemia, and skin problems. It is seen that most of the respondents had more than one health problem. Osteoarthritis was found to be more common among females while other health problems were almost similar among both the genders.

**Table 2:** Morbidity pattern of the respondents

Diseases	Males	Females	Total
Hypertension	53 (57.6)	73 (60.3)	126 (59.1)
Diabetes	11 (11.9)	11 (9.0)	22 (10.3)
Osteoarthritis	19 (20.6)	69 (57.0)	88 (41.3)
Bronchial asthma	13 (14.1)	10 (8.2)	23 (10.7)
Others	17 (18.4)	19 (15.7)	36 (16.9)

Figures in parentheses are in percentages

### Attitudes towards old age

Table 3 shows that almost 98% of the respondents felt that old age had affected their day-to-day life. Among these, 86.4% felt that age had partially affected their daily activities. Half of the people interviewed felt neglected by their family members, while 47% felt unhappy in life and 36.2% felt they were a burden to the family. An unfavorable attitude was observed to be more among females than males.

**Table 3:** Attitude towards old age

	Males	Females	Total (n=213)
Old age has affected day-to-day life	88 (42.3)	120 (57.7)	208 (97.7)
Partially	76 (41.3)	108 (58.7)	184 (86.4)
Completely	12 (50.0)	12 (50.0)	24 (11.4)
Feel neglected by family members			
Always	4 (40.0)	6 (60.0)	10 (4.7)
Sometimes	46 (37.7)	76 (62.3)	122 (57.3)
Feel a burden to family	34 (44.2)	43 (55.8)	77 (36.2)
Not happy in life	46 (45.0)	56 (55.0)	102 (47.9)
Feel they are not loved by family members	28 (35.9)	50 (64.1)	78 (36.6)

Figures in parentheses are in percentages

Table 4 shows that females had poor perception regarding economic and social security as compared with males. Approximately 40% of the respondents interviewed had feelings of insecurity while around 56.3% were deprived of financial security. Other reasons of insecurity included illness, not having issues or male children.

**Table 4:** Perceptions of elderly regarding economic and social security

Perceptions regarding security	Males (%)	Females (%)	Total (%)
Deprived of finances	47 (39.2)	73 (60.8)	120 (56.3)
Deprived of companions	4 (28.6)	10 (71.4)	14 (6.6)
Troubled with feelings of insecurity	35 (41.1)	50 (58.9)	85 (39.9)

Table 5 shows that 48% of the respondents felt sad mainly because of poverty followed by illness (41.3%). Other reasons for feeling sad were unwed daughters at home, alcoholic son/son-in-law, financial loss, illness of spouse, children staying away from them, death of children, or not owning a house.

**Table 5:** Reasons for feeling sad

Reasons	No. (n=213)
Poverty	102 (47.9)
Illness	88 (41.3)
Neglected	28 (13.1)
Loss of spouse	22 (10.3)
Loneliness	11 (5.2)
Others	54 (25.4)
Daughters not married	14 (6.6)
Alcoholic son/son-in-law	11 (5.2)
No issues or no male issues	4 (1.9)
Illness of the spouse/children	8 (3.8)
Children staying away	8 (3.8)
Financial loss	3 (1.4)
Death of children	3 (1.4)
Not owning a house	3 (1.4)

Figures in parentheses are in percentages

It was also observed in the study that only 35.7% were aware of the government welfare schemes for the elderly and only 14.6% (31) had utilized the geriatric welfare services in our study. Three-fourths of the population studied was not eligible for these schemes because of having male children or property.

It was observed that 68.5% of the respondents had friends and social contacts outside the home. In case of a conflict with family members, nearly half of the respondents (45%) preferred to sleep in order to get over it, 33% preferred to discuss it with others, and 20% preferred to find a solution.

It was observed in our study that around 52% of the respondents felt that old age affected their role in the family. A total of 35% of the respondents felt they were not consulted by the family members for making decisions. They felt they were ignored by family members because of their physical illness and economic dependence. In spite of being unhappy due to these problems, they still preferred their home to an old age home for their residence.

## Discussion

Almost more than half of the respondents who were interviewed were from joint families (56.8%), while 33% were from a nuclear family. Various studies by Padda, *et al.*,(6) Singh,*et al.,*(7) and Sivamurthy, *et al.*(8) have brought out similar findings. The higher prevalence of joint families could be because of the rural study area and social migration of the youngsters being less when compared with cities.

It is indeed true that it is the marital status that determines ones position within the family as well as the status in society. The proportion of elderly married, widowed, or unmarried were found to be similar to the study conducted by Singh, *et al*.(7) Shah(9) reports that 64.3% of elderly women were widows and most of them were dependent.

According to the NSS 52^nd^ round,(10) 63% of the elderly were illiterate in India. Our study showed that almost half of our respondents were illiterate and around 37% had education upto the primary level. Padda,*et al.*(6) reported 38.6% illiteracy at Amritsar, while it was 78% in a study conducted in Tamil Nadu by Elango,(11) and Singh, *et al.*(7) reported 80.2%.

It is observed in this study that illiteracy is higher among females (62%) than males (22.8%). The disparity in literacy status may be attributed to the area being rural. Educating females in those days was not considered as important as establishing a marriage at an early age.

In our study, approximately 18.7% were still working as unskilled workers against those who were at home (78%). Similar results were seen in a study by Elango,(11) while Singh, *et al.*(7) in his study, reported that 55.8% were occupied in productive work, 28% in agriculture, 15.1% in labor, and 44.2% were dependent on others.

Half of the interviewed subjects felt neglected by their family members unlike in the study conducted by Singh, *et al.*,(7) which reported that 26.1% felt neglected by family members, while Prakash, *et al.*(12) reported 17.3% having feelings of neglect.

In this study, 47.9% of the respondents said that they were not happy in life as compared with 53.2% reported by Singh, *et al*.(7) A total of 68.5% of the respondents said they had friends and that they participated in social functions. Half of them would visit their neighbors or relatives. Goel, *et al.*(13) reported that 24.8% were not having any social contact outside the home as compared with approximately 32% in our study. Almost half of the respondents felt neglected and sad and felt that people had an indifferent attitude towards the elderly as compared with 8.9% reported by Singh,*et al.*. Some of the respondents thought that people don't respect them because they were aged and could not contribute to the family and society.

Around 48% of the respondents mentioned that they felt sad mainly because of poverty followed by illness (41.3%). Unlike our study, Singh, *et al.*(7) reported that the main reason for feeling sad was loneliness (20%), followed by neglect in the family (26.1%), illness (11.5%), and economic causes (10.2%). Prakash *et al.*(12) reported that 23.3% of the respondents felt sad because of loneliness followed by feeling neglected (17.3%). In his study, Goel, *et al.*(13) mentioned that 55.1% of the respondents had a negative attitude towards life.

A study conducted by Goel, *et al.*(13) showed 45% of the respondents had utilized geriatric welfare services as compared with 14.6% in our study. In our study, 52.6% of the respondents felt that old age affected their role in the family as compared with 38% in the study conducted by Elango.(11) It was observed in our study that even though the respondents were not very happy in life or did not have a good relationship with their children, they still preferred to stay at home or die rather than stay in old age home.

## Conclusions

The results of this study showed that a major proportion of the elderly were out of the work force, partially or totally dependent on others, and suffering from health problems with a sense of neglect by their family members. There is a growing need for interventions to ensure the health of this vulnerable group and to create a policy to meet the care and needs of the disabled elderly. Further research, especially qualitative research, is needed to explore the depth of the problems of the elderly.

The authors do accept some of the limitations of this study. A scoring system for attitude was not used because the objective of the study was not to quantify the attitudes and grade the subjects but just to assess the load of existence of such problems among the subjects. Since the subjects included in the study were patients attending the clinics with various health problems, the study findings cannot be generalized to the community at large.
